# Attachment Patterns Trigger Differential Neural Signature of Emotional Processing in Adolescents

**DOI:** 10.1371/journal.pone.0070247

**Published:** 2013-08-05

**Authors:** Maria Josefina Escobar, Alvaro Rivera-Rei, Jean Decety, David Huepe, Juan Felipe Cardona, Andres Canales-Johnson, Mariano Sigman, Ezequiel Mikulan, Elena Helgiu, Sandra Baez, Facundo Manes, Vladimir Lopez, Agustín Ibañez

**Affiliations:** 1 Laboratory of Experimental Psychology and Neuroscience (LPEN), INECO (Institute of Cognitive Neurology) and Institute of Neuroscience, Favaloro University, Buenos Aires, CABA, Argentina; 2 National Scientific and Technical Research Council (CONICET), Buenos Aires, CABA, Argentina; 3 Laboratory of Cognitive and Social Neuroscience (LaNCyS), UDP-INECO Foundation Core on Neuroscience (UIFCoN), Diego Portales University, Santiago, RM, Chile; 4 Escuela de Psicología, Pontificia Universidad Católica de Chile, Santiago, RM, Chile; 5 College of Psychology, Harvard University, Cambridge, Massachusetts, United States of America; 6 Department of Psychology and Department of Psychiatry and Behavioral Neuroscience, The University of Chicago, Chicago, Illinois, United States of America; 7 Departamento de Física, FCEN, Universidad de Buenos Aires and IFIBA, CONICET, Buenos Aires, CABA, Argentina; 8 Universidad Torcuato Di Tella, Buenos Aires, CABA, Argentina; 9 Medical Research Council (MRC), Cognition and Brain Sciences Unit, Cambridge, United Kingdom; University of Granada, Spain

## Abstract

**Background:**

Research suggests that individuals with different attachment patterns process social information differently, especially in terms of facial emotion recognition. However, few studies have explored social information processes in adolescents. This study examined the behavioral and ERP correlates of emotional processing in adolescents with different attachment orientations (insecure attachment group and secure attachment group; IAG and SAG, respectively). This study also explored the association of these correlates to individual neuropsychological profiles.

**Methodology/Principal Findings:**

We used a modified version of the dual valence task (DVT), in which participants classify stimuli (faces and words) according to emotional valence (positive or negative). Results showed that the IAG performed significantly worse than SAG on tests of executive function (EF attention, processing speed, visuospatial abilities and cognitive flexibility). In the behavioral DVT, the IAG presented lower performance and accuracy. The IAG also exhibited slower RTs for stimuli with negative valence. Compared to the SAG, the IAG showed a negative bias for faces; a larger P1 and attenuated N170 component over the right hemisphere was observed. A negative bias was also observed in the IAG for word stimuli, which was demonstrated by comparing the N170 amplitude of the IAG with the valence of the SAG. Finally, the amplitude of the N170 elicited by the facial stimuli correlated with EF in both groups (and negative valence with EF in the IAG).

**Conclusions/Significance:**

Our results suggest that individuals with different attachment patterns process key emotional information and corresponding EF differently. This is evidenced by an early modulation of ERP components’ amplitudes, which are correlated with behavioral and neuropsychological effects. In brief, attachments patterns appear to impact multiple domains, such as emotional processing and EFs.

## Introduction

Research suggests that individuals with different attachment patterns process social information differently, especially in terms of facial emotion recognition. [1]–[7]. Nevertheless, few studies have examined the neural systems involved in facial emotion for different attachment patterns [8]. To our knowledge no study has explored the neural correlates of attachment patterns in adolescents. Adolescence is a crucial life stage in the development of the social brain [9] where significant changes at the emotional, cognitive, and behavioral level occur. These changes have been associated attachment patterns that reflect the transition to a self-sufficient individual instead of depending on others [10]. It is likely that attachment patterns in adolescents shape social information processing, especially facial emotion. Consequently, we posit that these processes should be reflected in neurophysiological and neuropsychological measures. The purpose of this study is to identify the cortical markers of emotion processing in adolescents with different attachment patterns and to explore their relation to individual neuropsychological profiles.

According to attachment theory, attachment orientations are represented as internal working models (IWMs) [11]. The IWMs of attachment influence the way people organize their behavior, including how they perceive, attend to, and process information of emotional significance [4]. Non-verbal interactions, especially facial expressions, are integral to attachment communication [11], [12]. The attachment system is based on a basic need for security and protection, and is activated in response to distress or threat. Individuals who present a secure attachment pattern have IWMs of their parents as available and responsive [13]. In contrast, an insecure attachment pattern stems from caregivers with an unavailable or unpredictable response to a child’s needs. Based on these concepts, Ainsworth [14] proposed a classification of three attachment patterns: one secure attachment pattern (described above) and two insecure attachment patterns. The insecure-ambivalent/anxious pattern encodes IWMs of their caregivers as unpredictable individuals. Thus, the child seeks to remain near the caregiver to increase chances of contact. Individuals with an insecure-avoidant pattern have IWMs that depict the caregiver as consistently failing to provide security [15], [16]. Expanding on Ainsworth’s research, Main and Solomon [17] defined a disorganized pattern of attachment, in which individuals have IWMs that represent their caregivers as a possible threat, causing the child to adopt to fearful or disoriented behavior [15], [18]. Thus, it is possible that the type of IWMs in attachment could explain some differences in the processing social cognitive information [19].

A secure attachment pattern has been correlated with numerous benefits to an individual’s psychological well-being beyond the inter-subjective and social domain. A primary caregiver’s consistency and availability enable a child to freely explore the environment and increase a child’s confidence in receiving comfort. Furthermore, these early experiences influence cognitive abilities, such as attention and memory processes for attachment-relevant information [20], [21]. For example, insecure attachment is associated with reduced attention to angry faces, which can reflect a failure to notice threatening stimuli [21]. In terms of memory, studies have found that insecure individuals can suppress attachment-relevant information that would cause emotional pain, while secure individuals process their attachment-relevant information fully and flexibly [15]. Furthermore, a relationship between attachment and general cognitive abilities has been observed in some studies. For instance, individuals with secure attachment perform better academically [22]–[24]. Moreover, an association has been evidenced between performance on general attention tasks and attachment style [25]. For example, the latter study reported that avoidant individuals regulated their attention mainly by ignoring potential distracters. Furthermore, research suggests that secure attachment is associated with high performance on executive function tasks, (EF) [26] such as increased language competence [27]. These findings suggest that general cognitive performance and cognitive abilities, such as attention and memory for attachment-relevant information, are correlated with different attachment patterns.

Recent empirical evidence also demonstrates that individuals process facial emotional information based on attachment style [1], [2], [4]–[7]. In neuroimaging studies, individuals with insecure (avoidant or anxious) attachment exhibited differential modulations of neural responses to facial expressions than individuals with secure attachment [2], [6], [28]. Moreover, individuals with avoidant attachment showed a weaker activation of the somatosensory cortex to sad, masked faces [6] and lower activation of the ventral striatum and ventral tegmental areas in response to smiling faces followed by positive feedback [28]. These results suggest the existence of a tendency for avoiding negative emotional states that demand attachment-system activation [6], [7] and positive social signals [28]. Anxious attachment was demonstrated to be positively related to activation of the left inferior, middle, and medial prefrontal areas, and globus pallidus, claustrum, and the right cerebellum in response to masked happy faces [2]. Moreover, anxious attachment has been associated with increased activation of the left amygdala in response to angry faces followed by negative feedback [28]. These studies indicate that individuals with anxious attachment are more responsive to emotional facial signals at an automatic processing level than are individuals with secure attachment [2], [28].

Processing of emotional information in faces has been extensively studied using event-related potentials (ERPs) [29]. This technique provides excellent temporal resolution for assessing cognitive brain processes. Current ERP research in social neuroscience highlights the role of early and late cortical dynamics [29]. Early responses (e.g., 80–200 ms after stimulus onset) usually index bottom-up sensory mechanisms sensitive to stimulus. For instance, early modulation refers to the facilitation of early automatic and pre-attentional discrimination of salient stimuli. Later stages (300–800 ms) may reflect top-down control mechanisms that influence the processing of task-relevant stimuli. The late process can be interpreted as correlates of arousal, control, and awareness. Nevertheless, early components, especially the N170, have evidenced modulation through different top down mechanisms. Examples include ingroup bias [30], attention [31], and awareness [32]. Moreover, the N170 emotional modulation is a good predictor of social-cognitive profile (executive functions, processing speed, fluid intelligence and theory of mind) in normal as well as psychiatric conditions [33]. To our knowledge, few studies have explored the relationship between attachment orientation and emotional face-processing using early ERPs. It is important to note that all of these studies have shown differences in the modulation of components among adult attachment styles. Because previous research on attachment has focused on late components, assessing the N170 modulation would expand the literature by providing a measure of early and automatic processes influenced by top-down effects. For the current study we reported the P1 and N170 components.

The P1 and N170 ERP components are especially useful for examining individual differences between attachment orientation and emotional face-processing. The P1 component can be modulated by the stimulus type (ST), which is elicited by comparing faces to words [34], [35]. For instance, significant differences in the P1 component in response to angry face stimuli compared to neutral stimuli have been observed in individuals with avoidant attachment [36]. This difference was not present in secure individuals or anxious individuals. Furthermore, the N170 is an early cortical response that is triggered more strongly with facial stimuli, as compared to object or word stimuli [34], [37]. To our knowledge, only one study has assessed facial processing indexed by N170 for different attachment patterns [3]. Insecure women showed a more pronounced negativity in the face-sensitive N170 component. The authors concluded that encoding faces was more challenging for insecure-avoidant women than for secure-attachment women, as insecure-avoidant women showed greater activation of cortical and processing resources. In general, the main finding in these studies, amplitude modulation of known ERP components [3], [36], [38], suggests that differences in attachment patterns are related to differences in facial emotion processing.

Studies that have examined the brain areas involved in the perception of facial emotion among attachment styles, have used adult populations [3], [36], [38]. To our knowledge, no study on attachment style has focused on adolescents. Since adolescence marks a crucial stage in the social brain development, studying attachment style during this life stage is an important area of research [9]. The current study aims to explore the brain correlates of emotional information processing in adolescents with different attachment patterns. We also sought to determine the relation of attachment patterns to the neuropsychological profile of adolescents.

The primary aim of this study was to assess whether there exists an association in adolescents between attachment patterns and capability to process emotional facial expressions. To address this question we chose an ERP design based on a modified version of the dual valence task (DVT) [39], [40]. Participants had to classify stimuli according to its emotional valence (positive or negative). Faces and words were presented to test the effects of ST (faces vs words) and valence (positive vs negative). Our second aim was to explore whether the attachment patterns were related to individual neuropsychological profiles. Consequently, participants were required to undergo a comprehensive neuropsychological assessment.

Based on these antecedents, we hypothesized that: 1) Participants with different attachment patterns will show variations in emotional processing, as indexed by a differential modulation of ERP amplitudes while viewing face stimuli; 2) Individuals with insecure attachment will exhibit larger amplitudes in the P1 and in the N170 in response to face stimuli and exhibit a differential modulation of emotional valence; 3) Groups varying in attachment pattern will also differ at the neuropsychological level; improved performance is expected for the secure attachment group.

## Methods

### Ethics Statement

Participants and their parents read and signed an informed consent in agreement with the Declaration of Helsinki before beginning the study. The ethical committee of the Psychology Faculty, Pontificia Universidad Católica approved the study.

### Participants

The present study is part of the Attachment Adoption Adolescents Research Network (AAARN), an international project focusing on attachment representation in adolescents and their parents. Participants were recruited from several sources, such as social networks (Facebook groups, chain letters) and institutions [S*ervicio Nacional de Menores (SENAME), Fundación Chilena para la Adopción and Fundación San José*]. The final sample consisted of 40 adolescents between 11 and 16 years of age. After the child’s neuropsychological evaluation, parents were offered a copy of the report. The sample included two groups: adolescents with secure attachment (SAG) and adolescents with insecure attachment (IAG). In both groups, some participants (6 for SAG and 8 for IAG) presented late adoption history (after 6 months). As requested by one reviewer, we covariate all results (behavioral and ERP measures) with age of adoption. No no significant effect of covariance were observed.

A semi-structured interview, the *Friends and Family Interview* (FFI) [41], was used to evaluate the representations of adolescent attachment patterns. The FFI has 8 dimensions, each one with several subcomponents: coherence, truth, economy, relation, manner and overall coherence; reflective function [developmental perspective, theory of mind (mother, father, sibling, friend and teacher), and diversity of feelings (mother, father, sibling, friend, and teacher)]; evidence of secure base (father, mother, other significant figure); evidence of self-esteem: social and school competence; peer relations (frequency and quality of contact); sibling relations (warmth, hostility and rivalry); anxieties and defenses [idealization (self, mother and father), role reversal (mother and father), anger (mother and father), derogation (self, mother and father) and adaptive response]; and differentiation of parental representations. The interview also contains a non-verbal code to evaluate fear/distress and frustration/anger and contains a global attachment classification. The assessments are scored on a 4-point Likert scale (1 = *no evidence* and 4 = *marked evidence*) [42].

Four global attachment categories were used in this study: secure attachment, insecure-dismissing attachment, insecure-preoccupied attachment and disorganized attachment. The duration of each interview averaged 35 minutes (minimum of 18 minutes and maximum of 1 hour 40 minutes). Every interview was video-recorded and transcribed. Interviews were coded using both video and transcription materials. To assess for potential interviewer bias, two trained evaluators coded 6 interviews, which had a Cohen’s Kappa = 0.94. A trained evaluator coded the other 44 interviews. The validity of this measure as an indicator of security and organization of attachment has been previously tested and confirmed across countries [43].

The final sample included 20 secure (50%), 15 insecure-dismissing (37%), and 5 insecure-preocupied (13%) participants (none were disorganized). Due to the small sample size, the insecure-dismissing and insecure-preocupied attachment styles were combined into a single “insecure attachment group” following previous research methods [22], [23]. The IAG (n = 20; mean age = 12.15 years, SD = 1.26) was contrasted with the SAG (n = 20; mean age = 13.10 years, SD = 1.29). The IAG consisted of 13 males and 7 females, and the SAG consisted of 9 males and 11 females. We controlled for between group differences in age (F(2, 37) = 0.22, p = 0.81), sex (X^2^(2) = 1.81, p = 0.40), and education level (F(2, 37) = 1.54, p = 0.22). Participants had no history of physical or mental disorders, according to institutional records and a neuropsychiatric interview with the parents. Participants along with their parents gave informed consent in agreement with the Declaration of Helsinki. The Ethics Committee of the Institute of Cognitive Neurology approved all experimental procedures.

### Instruments

#### Neuropsychological assessment

All participants completed a neuropsychological battery assessing attention, speed processing, visual-spatial abilities, and EF. In the verbal fluency task, participants were given a category or a letter and asked to state all of the words that came to mind in one minute. In the digit span subtest [44], participants were asked to repeat a given set of numbers in the same order (digit span forward) or in reverse order (digit span backward). The block design task [44] required participants to arrange cubes of red, white, or red and white sides to form a specific pattern. For the picture arrangement task [44] participants were required to piece together a misarranged story into the correct order. In the symbol search task [44], participants were asked to decide whether a given symbol was present in a line-up of other symbols. The coding subtest [44] required participants to decipher a numerical code using symbols. To measure attention and speed processing, we incorporated the trail making test [45], which entails connecting numbers in sequential order (test A) or letters and numbers (test B) spread out randomly on sheet of paper.

#### Emotional processing

Dual Valence Task (DVT). The DVT [39], [46]–[48] is an adaptation of the Implicit Association Task designed specifically for ERP measurements [40]. The DVT assesses the emotional valence (positive or negative) of faces and words. Participants are asked to categorize words as either pleasant or unpleasant and faces as either happy or angry, and to make these judgments as fast and as accurate as possible. The DVT allows for behavioral measures through reaction time of responses and electrophysiological measures through activation of early ERP components. In our study, participants were presented with a series of four blocks on a computer screen: 3 practice blocks and one test block. Practice blocks used different face and word stimuli than test blocks. Trials began with a fixation cross presented for 1000 ms followed by the stimulus, which was shown for 100 ms. Immediately after, a fixation cross appeared on the screen and disappeared either after 2000 ms or the participant’s response, whichever came first. After a response, there was an interstimulus interval (ISI) of 1000 ms. Each stimulus was centered horizontally and vertically on the screen subtending a visual angle of 4.5°×3.15° at a viewing distance of approximately 80 cm. Eighty happy and angry facial expressions and 142 pleasant and unpleasant word stimuli were included. The happy and angry sets of pictures depicted the same people. Faces were previously controlled for arousal, valence, emotion (angry vs. happy), and physical properties, and words were controlled for arousal, valence, predictability, content, length, and frequency (for details see [49]).

#### Control variables

Family data form and history of adoption. Parents were questioned on socio-demographic family data (socioeconomic level, parent’s educational level, and child’s educational level), age at adoption, health history of child birth and subsequent complications, health information prior to the adoption, and the child’s medical or mental health history and current health information.

### Procedure

Once the family was contacted, participants and their parents signed a consent form. Next, an interview with the participant’s mother was conducted. The attachment interview with the participant took place later on. Interviews were administered at the participants’ homes. In the first session, participants were completed the neuropsychological battery in order to test general cognitive processes. Lastly, during the second interview (taken within 10 days) the electroencephalographic (EEG) was recorded while participants performed the DVT.

### EEG Recordings and Preprocessing

EEG signals were recorded with HydroCel Sensors from a GES300 Electrical Geodesic amplifier at a rate of 500 Hz using a system of 129-channels. Data that were outside a frequency band that ranged from 0.1 Hz to 100 Hz were filtered out during the recording. Later, the data were further filtered using a band-pass digital filter with a range of 0.3 to 30 Hz to remove any unwanted frequency components. During recording, the vertex was used as the reference electrode by default, but signals were offline re-referenced to average electrodes. Two bipolar derivations were designed to monitor vertical and horizontal ocular movements (EOG). Continuous EEG data were segmented during a temporal window that began 200 ms prior to the onset of the stimulus and concluded 800 ms after the offset of the stimulus. Eye movement contamination and other artifacts were removed from further analysis using both an automatic (ICA) procedure and a visual procedure. No differences were observed between groups regarding the number of trials. All conditions yielded a least 87% of artifact-free trials.

#### Region of Interest (ROIs)

Based on previous DVT reports [39], [46]–[48], ROIs were used to analyze the scalp topography of the ERP components. The ROIs were chosen by visual inspection of the right N170 component, comprised of four electrodes placed near the canonical locations for the N170 component (T6 and T7: [50]). Consequently, we included 4 electrodes (the canonical locations and 3 adjacent electrodes) for each hemisphere (left: 58, 59, 64, and 65; right: 90, 91, 95 and 96). We also performed an additional data-driven electrode choice on the basis of the maximum peak amplitude of the N170 component to confirm that the selected electrodes did in fact generate the N170 modulation. This is an expected result because the canonical locations of the N170 component (T6 and T7) and the electrodes that are adjacent to them often yield the maximum peak amplitude [50].

#### Mean amplitude

P1 measures were computed by using a fixed temporal window (90–130 ms), after which the mean amplitude of the P1 signal was obtained for the mean of each category and each subject. The same procedure was computed for the N170 at 140–190 ms time window. The ERP modulation that is observed in the DVT is very sensitive to mean amplitude and is not sensitive to latency [39], [40], [46], [51].

### Data Analysis

ANOVAs and Tukey’s HSD post-hoc comparisons (when appropriate) were used to compare the demographic, neuropsychological, and reaction time data across all of the groups. Repeated measures ANOVAs and Tukey’s HSD post-hoc comparisons (when appropriate) were performed to analyze the DVT and ERP data. Three within-subjects factors, stimulus type (ST: faces vs words) and two valences scores (separately for each stimuli, face valence and word valence: positive vs negative), were included. One between-subjects factor with 2 levels was considered (group: SAG, IAG). The Matlab software program and the EEGLab toolbox were used for the offline processing and analysis of the EEG data. Finally, global scores of significant between-group effects (ST: face-minus-word) at P1 as well as face (total score) and face valence (face positive and face negative at left and right hemisphere) at N170 were correlated with the neuropsychological performance of participants.

## Results

### Neuropsychological Assessment

The SAG performed better than the IAG on coding (F(1, 38) = 11.45, p<0.01), block design (F(1, 38) = 7.10, p<0.05), and Trail Making Test B (F(1, 38) = 4.86, p<0.05). A trend for significance was observed on the digits (F(1, 38) = 3.16, p = 0.08) and symbol search (F(1, 38) = 3.78, p = 0.06) tasks, with the SAG scoring higher than the IAG. No significant differences between groups were observed on the verbal fluency task, picture arrangement task, or Trail Making Test A. See Table 1.

**Table 1:** Neuropsychological assessment.

	SAG		IAG		SAG vs. IAG
	*M*	*SD*	*M*	*SD*	
**Neuropsychological Assessment**					
Picture Arrangement	23.65	6.05	22.10	8.36	NS
Cube Construction	46.85	9.48	38.30	10.78	**0.01**
Symbol Search	26.40	6.31	23.05	4.41	**0.06**
Digits	12.05	3.35	10.40	2.46	**0.08**
Verbal Fluency	16.08	3.45	14.75	3.90	NS
TMTA	44.10	11.57	47.25	11.72	NS
TMTB	96.50	23.30	126.55	56.31	**0.04**
Coding	54.10	9.21	45.55	6.55	**0.01**

### DVT (Behavior)

#### Stimulus type

A main effect of ST (F(1, 38) = 27.74, p<0.01) evidenced that participants performed better on face stimuli recognition than word stimuli recognition. A main effect of ST (F(1, 38) = 22.75, p<0.01) was also observed for reaction time, indicating that participants responded faster to face stimuli than word stimuli. In addition, an effect of group (F(1, 38) = 4.05, p<0.05) revealed that the IAG had slower reaction times than the SAG.

#### Valence effects

An interaction between valence × group was significant (F(1, 38) = 6.30, p<0.05). Post-hoc comparisons (Tukey HSD MS = 57863, df = 52.36) revealed that participants in the IAG tended (p = 0.06) to respond slower to negative words than participants in the SAG. See Table 2.

**Table 2:** DVT behavioral measures.

	Accuracy (%)			
	SAG		IAG	
Category	M	SD	M	SD
Face	86.59	11.21	83.91	12.33
Word	81.75	12.13	76.06	15.99
Face Negative	87.62	11.23	84.31	14.64
Word Negative	81.38	12.93	75.56	15.38
Face Positive	85.56	12.17	83.56	12.24
Word Positive	82.12	12.25	82.12	76.56
	**RT (ms)**			
	**M**	**SD**	**M**	**SD**
Face	707.51	126.83	789.20	205.57
Word	873.00	201.07	988.92	237.30
Face Negative	700.87	108.86	807.81	232.05
Word Negative	819.78	239.74	1013.05	240.63
Face Positive	714.14	166.85	770.59	216.25
Word Positive	926.23	180.09	964.80	289.21

### DVT (ERPs)

**Figure 1:**
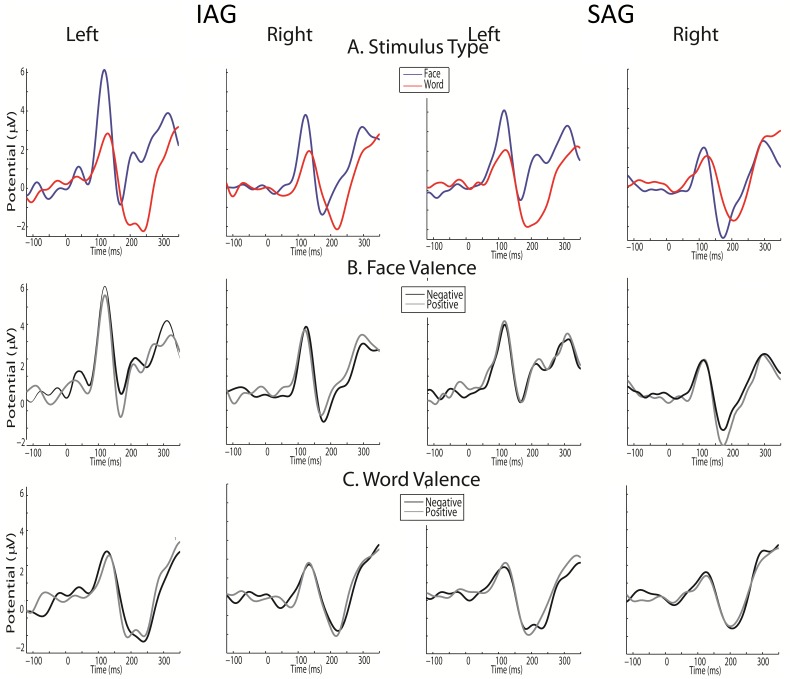
P1 and N170 results. A) Stimulus type (ST) effects at left and right hemispheres for both groups. B) Face valence (FV) effects at left and right hemispheres for both groups. C) Word valence (WV) effects at left and right hemispheres for both groups. IAG: Insecure attachment group. SAG: Secure attachment group.

Figure 1 shows the P1 and N170 effects for both groups and conditions.

#### P1 effects

**Figure 2:**
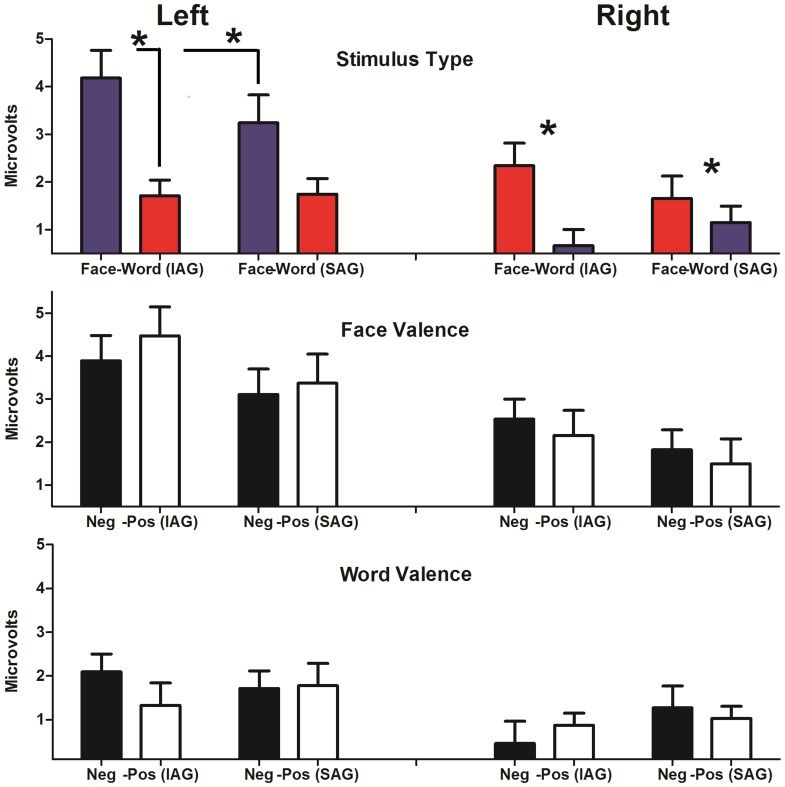
Mean amplitude values for P1. A) Stimulus type (ST) effects at left and right hemispheres for both groups. B) Face valence (FV) effects at left and right hemispheres for both groups. C) Word valence (WV) effects at left and right hemispheres for both groups. Asterisks indicate significant differences. IAG: Insecure attachment group. SAG: Secure attachment group.

A main effect of ST (Face>words; F(1, 38) = 37.03, p<0.001) and hemisphere (left>right, F(1, 38) = 12.37, p<0.001) evidenced an early facilitation of faces and left hemispheric dominance. Differences among groups (ST × group F(1, 38) = 4.49, p = 0.04) followed by post hoc interactions (MSE = 2.11, df = 65.71) revealed that faces elicited higher amplitude in the IAG than the SAG (p<0.05). ST effects in both groups evidenced also a face dominance (face>word; IG: p<0.0001; SG: p<0.05). See figure 2A.

The same effect of hemisphere (left>right; F(1, 38) = 9.30, p<0.005) was observed for face valence (FV). No other effects were observed (figure 2B).

As for face valence, hemisphere modulated the P1 elicited by word valence (WV; left>right; F(1, 38) = 14.93, p<0.001). No other significant results were observed (figure 2C).

#### N170 effects

**Figure 3:**
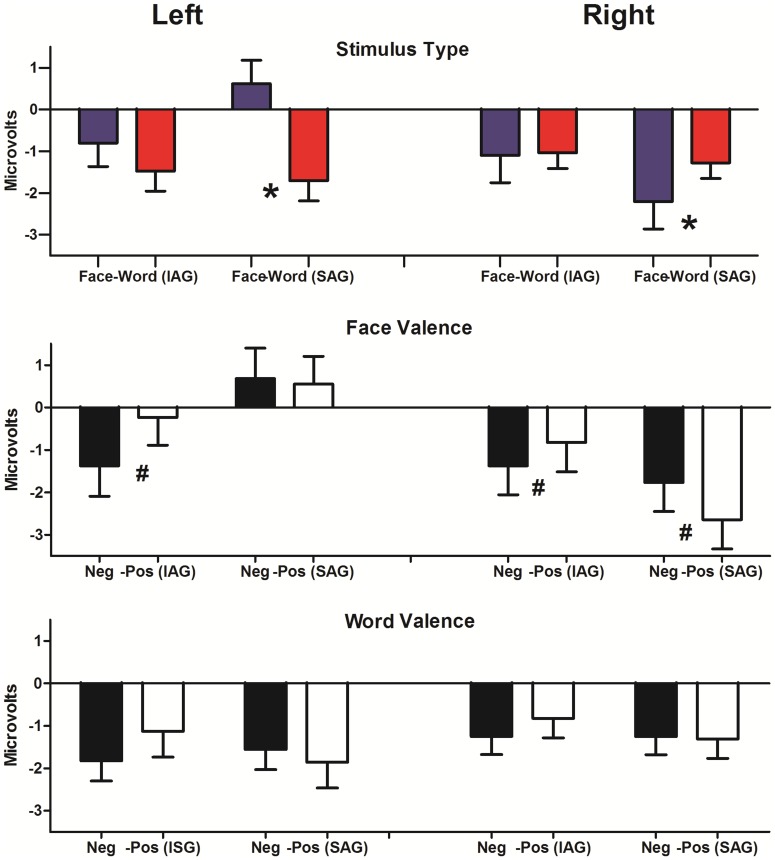
Mean amplitude values for N170. A) Stimulus type (ST) effects at left and right hemispheres for both groups. B) Face valence (FV) effects at left and right hemispheres for both groups. C) Word valence (WV) effects at left and right hemispheres for both groups. Asterisks indicate significant differences. IAG: Insecure attachment group. SAG: Secure attachment group.

A hemisphere × ST interaction (F(1, 38) = 9.17, p<0.005; post hoc Tukey HSD MSE = 8.62, df = 38.00) evidenced a left lateralized effect for semantic (words>face; p<0.05) and a non-significant right effect for facial processing (face>word; p = 0.71). Also, hemisphere × group interaction (F(1, 38) = 4.32, p<0.05), followed by post hoc comparisons (Tukey HSD, MSE = 3.37, df = 63.23) evidenced significant hemispheric (right>left) differences in the SAG only (p<0.05) but not in the IAG. Finally, a trend of hemisphere × ST × group (F(1, 38) = 3.67, p = 0.053, post hoc Tukey HSD MSE = 7.05, df = 66.02) indicates that in the SAG, a right face dominance (face>word, p<0.05) and a left word dominance (word>face; p<0.05) were significant (figure 3A).

Regarding face valence (FV), an interaction of hemisphere × group (F(1, 38) = 7.82, p<005; post hoc Tukey HSD, MSE = 7.43, df = 63.16) revealed a right dominance (right>left) in the SAG only (p<0.001). Finally, a trend of valence × group × hemisphere (F(1, 38) = 3.37, p = 0.06) followed by post hoc comparisons (MSE = 6.73, df = 55.81) evidenced valence effects (positive>negative) at right hemisphere in the SAG (p<0.05). Conversely, the IAG presented the opposite valence effect (negative>positive) at left (trend: p = 0.08) and right hemispheres (p<0.05). See figure 3B.

Finally, for word valence (WV), no significant effects were observed at N170 window (figure 3C).

### Correlations

**Figure 4:**
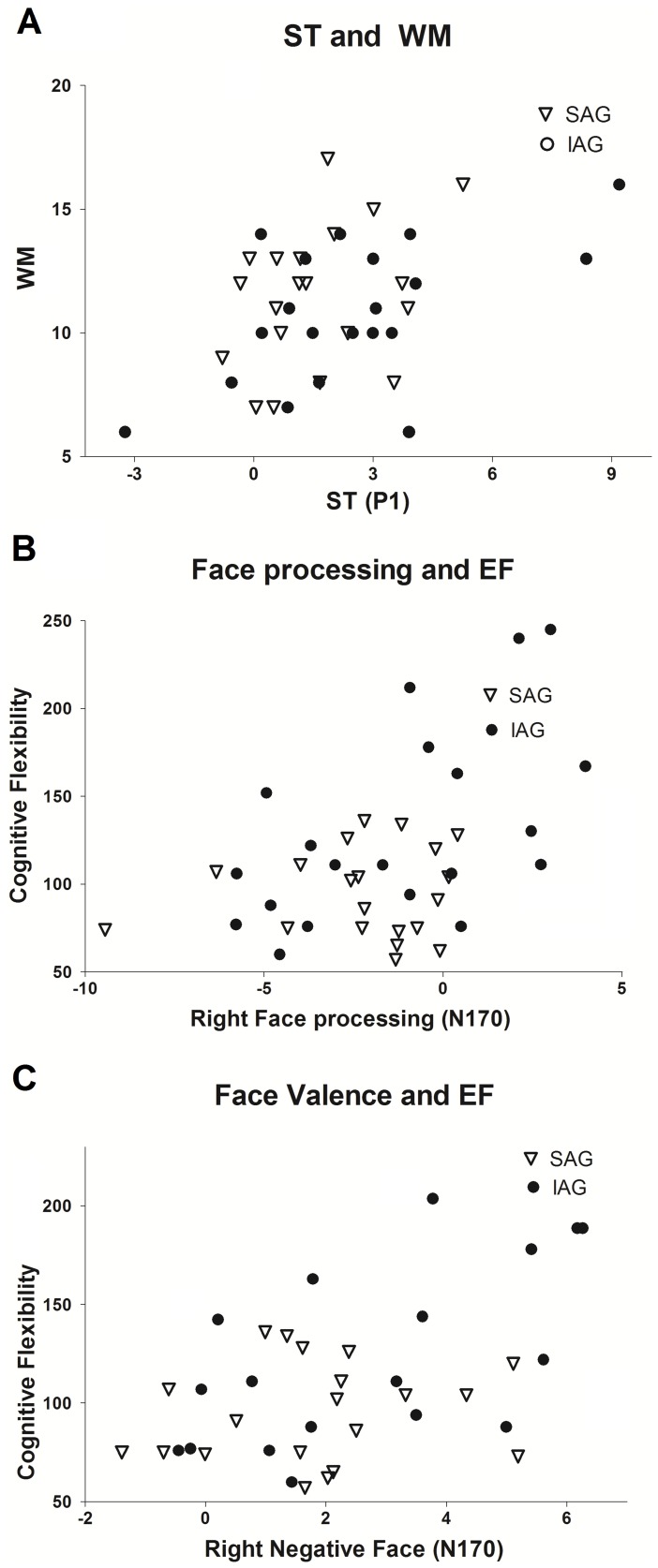
Association between individual differences and ERP results. A) ST at P1 and WM performance. B) Right hemisphere face processing (enhanced when more negative) correlated with cognitive flexibility. C) Face negative valence associations with cognitive flexibility at right hemisphere. D) Split analysis of IAG presented association between face negative valence and cognitive flexibility TMTB. IAG: Insecure attachment group. SAG: Secure attachment group.

Global scores of significance between-group effects (ST at P1; face and face valence at N170) were correlated with the neurocognitive profile of participants. Figure 4 lists the correlations for both groups.

#### P1

Enhanced ST discrimination at P1 was correlated with better WM performance (r = 0.32; p<0.001, figure 4A).

#### N170

Right hemisphere face processing (enhanced when more negative) was correlated with reaction times of cognitive flexibility (r = 0.37, p<0.001; figure 4B). Right hemisphere negative face valence was also associated with reaction times of cognitive flexibility at (0.37, p<0.05, figure 4C). In addition, when a split analysis by group was performed, the IAG presented associations between negative-face valence and cognitive flexibility (TMTB) at left (r = 0.45, p<0.005) and right hemispheres (r = 0.45 p<0.005).

## Discussion

The aim of the present study was to examine the behavioral and ERP correlates of emotional processing in adolescents with different attachment orientations and to explore the potential relationship between behavioral and ERP correlates and neuropsychological profiles. Previous studies have evidenced these relationships in adults [3], [8], [19], [28], [36], [38], [52]. However, few studies have researched emotional processing in adolescents [53], [54]. These results expand on previous theories in developmental neuroscience and attachment. Moreover, these findings suggest that the attachment process impacts multiple cognitive domains, such as emotional processing and EF.

We confirmed our hypothesis that individuals with varying attachment patterns process emotional information differently. This observation is evidenced by an early modulation of ERP amplitude followed by behavioral and neuropsychological effects. In sum, early cortical markers of face processing diverged in IAG relative to the SAG. The IAG exhibited larger P1 for face stimuli and attenuated the N170 component over the right hemisphere, indicating that they did not differentiate between emotions. Contrasting the amplitude of the N170 between the IAG and the SAG elicited by word and valence stimuli evidenced a negative bias for the IAG. Finally, the amplitude of the N170 elicited by face stimuli was correlated with EF in both groups (and negative valence with EF in the IAG).

### Neuropsychological Findings

As predicted from previous reports, the SAG scored higher than the IAG on neuropsychological evaluations. The IAG performed significantly worse on measures of attention and processing speed. Moreover, the IAG had a lower performance on tests of visuospatial abilities and cognitive flexibility. These data are consistent with previous research suggesting that individuals with secure attachment style perform better than those with insecure attachment on EF tasks [26]. These results also correspond with previous findings on the relationship between maternal attachment and child attachment with EF [55]. Overall our results suggest that attachment experiences may influence cognitive abilities.

### Behavioral Measures of Emotion Processing

The IAG performed worse on behavioral measures of emotion processing as assessed by the DVT. The IAG exhibited poorer accuracy and slower RTs for negative valence. This result is consistent with previous studies demonstrating that insecure individuals were slower and less accurate at differentiating angry faces from neutral ones [3], [21], [36]. For example, Dan and Raz [36] found that only the avoidant attachment group demonstrated slower RTs for angry faces compared to neutral faces. Anxious individuals, on the other hand, had poorer accuracy when differentiating angry faces from neutral ones; this effect was not presented in avoidant or secure participants [36]. In the current study, the IAG consisted of 15 insecure-dismissing (avoidant-like pattern) adolescents and 5 insecure-preoccupied (anxious-like pattern) adolescents. Due to the small sample size, especially in terms of insecure–preoccupied individuals, we cannot make definitive conclusions on this topic. Nevertheless, this behavioral pattern reaffirms the relationship found in prior studies between attachment security and abnormal processing of emotional valence.

### Neural Signatures of Stimulus Type and Emotion

No significant differences between the groups and ST were found for electrophysiological measures. We observed an early amplitude modulation of visual P1 elicited for face stimuli compared to word stimuli, which is consistent with previous research [34], [35]. In particular, these two studies found a significant difference between P1 for words and P1 for faces, but the P1 elicited by faces was the same as that for stimuli similar in complexity. The authors concluded that these dissimilarities did not reflect specialization (i.e.: linguistic vs. non-linguistic), but rather low-level differences between stimuli (i.e.: spatial frequency or size). Moreover, P1 amplitude has also been affected by the amount of attentional resources dedicated to a visual stimulus [56]. In this report, the face-elicited P1 showed a significant group effect. In other words, the IAG exhibited larger P1 amplitudes than with SAG.

Furthermore, abnormal P1 components elicited by faces have been observed in clinical populations. For example, anxious individuals exhibit larger P1s than non-anxious individuals [57]. This effect, known as hypervigilance, has been observed in recent studies. For instance, adult individuals with atypical attachment were found to have greater arousal after viewing scenes with negative emotional content [8], [19], [28], [36], [58], [59]. In our study, face stimuli elicited larger P1 for the IAG compared to the SAG in the left hemisphere. Nevertheless, different emotions were undistinguishable within this time window. In this context, we interpreted a larger face-elicited P1 in the IAG to indicate (a) a general state characterized by higher vigilance or (b) less efficient early structural face processing. Given that no valence differences were observed in the P1, alternative (b) seems to be the more likely explanation. However, further research is needed before any conclusion can be drawn.

In our study, the observation of a larger N170 for the SAG matched previously reported effects of ST [34], [50] and valence [40], [60]. Specifically, larger right N170 was observed for faces than for words, and larger N170 for positive compared to negative valence was detected. For the IAG, the ST effect at this time window was absent. This impaired discrimination at the N170 window could be interpreted as difficulty in semantic access. Supporting this claim, a meta-analytic study [27] showed that attachment styles were correlated with language abilities. The development of verbal capabilities and the use of language are closely related to the way children connect to their caregivers. Moreover, adults with insecure attachment exhibit greater difficulty in semantic processing of emotional faces than secure adults, which has been demonstrated by smaller N400 amplitudes during the presentation of emotion types [38]. In the present study, the impaired discrimination observed in the IAG suggests that the semantic skills learned in early relationships are maintained throughout adolescence.

As mentioned, the ST effect is also characterized by a lateralization in the right hemisphere, with a larger amplitude to face stimuli than to word stimuli [60]. In the present study this pattern was explicitly observed for the SAG. The IAG, however, showed abnormal right hemisphere activity within this time window. Previous reports on schizophrenia [47], bipolar disorder [48], and ADHD [39] have evidenced similar abnormalities in right hemisphere when assessing ERPs with the DVT. The impaired emotional processing indexed by N170 has been considered a useful biomarker of potential genetic deficits underlying these disorders. The presence of a similar pattern in our study raises the question whether potential environmental factors (i.e., attachment) modulate maturational pathways or whether a genetic predisposition independently causes this effect.

The N170 was larger in the IAG than in the SAG when viewing negative face stimuli. Previous studies have reported a similar negative bias in adult participants with insecure-avoidant attachment but at a different temporal window [52]. This finding stands in line with previous studies that have reported insecure individuals as more prone to a negative bias because they are more skilled at detecting threatening stimuli early and eliciting avoidant behaviors, [4], [36], [61], [62]. Moreover, poor quality face-to-face interactions, as described by Beebe et al. [63], may disrupt an adequate development of face affective processing. A bias for processing emotions accurately later on in life could be related to a difficulty in regulating emotions during early caregiver-child interactions. However, the N170 negativity bias is not specific to attachment patterns. It is also found in other populations with psychiatric disorders. For example, BD patients exhibited a negative bias at the N170 [48]. The presence of this bias in healthy adolescents with an insecure attachment pattern emphasizes the need to consider environmental and maturational factors in socio-emotional processing.

Previous research has suggested that facial and emotional processing involves parallel mechanisms that are partially dissociated over time [64]. Other studies have supported this claim. For instance, emotional N170 impairments were observed independent of deficits in facial structural processing [39]. In the present study, we found the IAG to have a deficient modulation of the N170 (reduced amplitude modulation of the N170 to faces compared to words). An abnormal modulation of negative facial emotion processing was also observed in the IAG.

In sum, adolescents in the IAG exhibited less efficient processing of negative-valence emotional information, particularly in faces. This effect was indicated by behavioral and electrophysiological measures. The IAG also exhibited an aberrant functional hemispheric lateralization that was less defined than in the SAG.

### Brain-behavior Associations

Electrophysiological measures were found to correlate with neuropsychological evaluations. EF (cognitive flexibility), particularly working memory (WM), was positively associated with the amplitude of P1 and N170. This P1, as previously stated, can be interpreted as attention allocation to stimuli [56], [65]. In other words, the greater the attention to external stimuli, the better the performance in WM tasks. The positive association between N170 amplitude and EF performance matches previous findings [33], [46]. For example, our study confirmed the association between secure attachment and performance in EF tasks [26]. Moreover, the IAG presented an association between negative valence and EF, which is consistent with current models of emotion-cognitive interactions [29], [66]–[68].

Compared with most attachment studies using ERPs, this report shows an early time window effect. The N170 plays an important role in indexing stimuli affected by top-down factors in a bottom-up fashion. Our results suggest that a relative automatic bias may be triggered by attachment patterns and may affect subsequent (later and controlled) cognitive processes.

Dramatic changes at both biological and psychological levels occur during adolescence. Studies have shown that important maturational changes in the social brain and developments in the face-processing areas of the brain also take place during this period [9], [69]. Several neurobiological, endocrine, and psychosocial variables are known to affect these processes. The findings in our study suggest that attachment style is an important factor in adolescence, because attachment is associated with emotion recognition and higher psychological functions such as EF, language, and socio-affective abilities [25]–[27], [70], [71]. Studies using adult participants have demonstrated the continuity of IWMs from adolescence into adulthood [72]–[74]. In addition, the present findings correspond with past research on adults and attachment orientations and provide new data on emotional information processing in adolescents. Furthermore, these findings can help fill the gap between different levels of analysis (socio-emotional, neuropsychological and electrophysiological) in adolescence.

### Limitations and Further Assessment

The present study has some limitations. First, our sample size is smaller than typical ERP studies on attachment styles in adults [3], [36], [38], [52]. Second, in an effort to gather a larger sample of participants with insecure attachment, we grouped two patterns of attachment into one, failing to distinguish between the types of insecure attachment (dismissive and preoccupied). Although this approach has been previously employed in other studies [22], [23], we could not detect whether the two attachment patterns affect social information processing differently. Previous studies in adults have found differences in the electrophysiological correlates of emotional processing between anxious and avoidant insecure individuals. As our study lacks statistical power, it is impossible to determine any differences in the insecure-preoccupied attachment pattern. Future studies should include the different insecure attachment patterns (insecure-dismissing, insecure-preoccupied, and disorganized).

## Conclusions

Confirming previous findings, the present study suggests that individuals with varying attachment patterns process facial emotional information differently [1], [2], [4]–[7], [28], and that these differences also affect other cognitive functions, such as EF [26]. Our study is the first to our knowledge to replicate these findings in adolescent populations. This study has several implications. First, it provides more in-depth understanding of the effects attachment patterns on social information processing, and adds to the knowledge on implementation of attachment patterns at the neural level (e.g., modulating the activity elicited by semantic and facial emotional stimuli). Second, this study emphasizes the importance of secure attachment in early life stages, as it may contribute to socio-emotional development in adolescence. Because adolescence involves seeking independence and distance from primary caregivers and a desire for new relationships, this life stage is crucial in the study of socio-emotional development. Furthermore, unforeseen environmental factors may affect the adoption of a particular attachment pattern. Consequently, thorough knowledge of relevant socio-affective and cognitive effects could aid in designing interventions that promote secure attachment. Finally, the present study contributes to the literature on adolescence, which has not been explored as thoroughly as other life stages.
